# Wellbeing, coping with homeschooling, and leisure behavior at different COVID‐19‐related lockdowns: A longitudinal study in 9‐ to 16‐year‐old German children

**DOI:** 10.1002/jcv2.12062

**Published:** 2022-02-11

**Authors:** Tanja Poulain, Christof Meigen, Wieland Kiess, Mandy Vogel

**Affiliations:** ^1^ LIFE Leipzig Research Center for Civilization Diseases Leipzig University Leipzig Germany; ^2^ Department of Women and Child Health University Hospital for Children and Adolescents and Center for Pediatric Research Leipzig University Leipzig Germany

**Keywords:** children, COVID‐19, homeschooling, longitudinal, media, physical activity, wellbeing

## Abstract

**Background:**

School closures are an effective measure against the spread of Covid‐19. However, they pose a major challenge to children, especially to those from socially disadvantaged families. The present study compared the wellbeing, coping with homeschooling, and leisure behavior of children and adolescents at two different periods of school closures in Germany. Wellbeing was also compared with wellbeing before the pandemic.

**Methods:**

Within the framework of the cohort study LIFE Child, 152 9‐ to 16‐year‐old children completed online surveys on wellbeing (KIDSCREEN‐27 scales on physical wellbeing, psychological wellbeing, and peer and social support), coping with homeschooling (concentration, motivation, fun, mastering of schoolwork, fear of bad marks), and leisure behavior (TV time, computer gaming time, indoor physical activity) during two COVID‐19‐related lockdowns in March 2020 (t1) and in January 2021 (t2). Data from both time points were compared using mixed‐effect models. Wellbeing was additionally compared with the wellbeing in 2019, before COVID‐19 (t0). We also assessed the effects of the socio‐economic status (SES) on all outcomes and changes between time points.

**Results:**

All considered wellbeing scores declined significantly between t0 and t1. Physical wellbeing decreased further between t1 and t2, while social support increased. Coping with homeschooling degraded significantly between t1 and t2, while leisure behavior did not change significantly. Lower SES was associated with lower physical wellbeing, poorer coping with homeschooling, longer computer gaming times, and a stronger decrease of concentration on schoolwork from t1 to t2.

**Conclusion:**

Repeated school closures have a negative effect on already compromised physical wellbeing and coping with homeschooling, especially in children from lower social strata.

## Graphical abstract

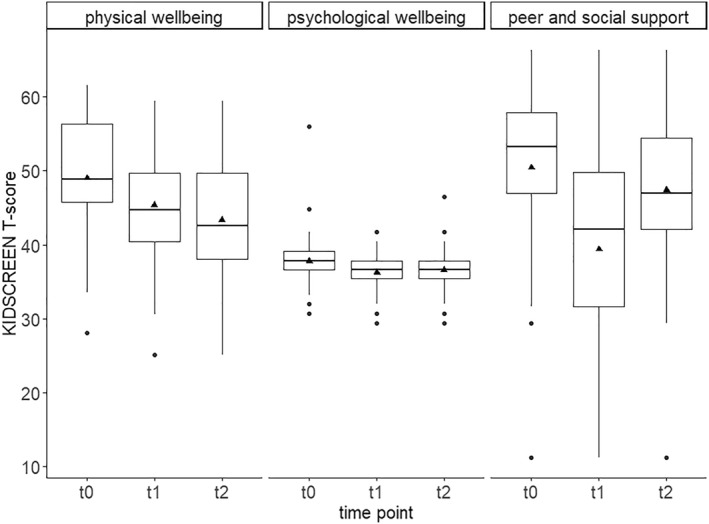

We assessed the wellbeing, coping with homeschooling, and lesirue behavior of 9‐ to 16‐year‐old German children and adolescents during two COVID‐19‐related lockdowns in spring 2020 and winter 2021. Compared to the pre‐pandemic period, physical wellbeing was lower during the first COVID‐19‐related lockdown and declined further from the first to the second lockdown. Similarly, coping with homeschooling was poorer during the second than during the first lockdown.

**Key points**

**What's known**

- Wellbeing of children is impaired by pandemic‐related school closures and contact restrictions.

**What's new**

- Compared to the pre‐pandemic period, physical wellbeing was lower during the first COVID‐19‐related lockdown (spring 2020) and declined further from the first to the second lockdown (winter 2021). Similarly, coping with homeschooling was poorer during the second than during the first lockdown.

**What's relevant**

- During a pandemic, each new school closure potentially worsens the already reduced wellbeing and school motivation of children and adolescents. Therefore, school closures should be prevented for as long as possible.

## INTRODUCTION

Since the beginning of 2020, we have been in a pandemic caused by the corona virus SARS‐CoV‐2. Economic and social life was shut down repeatedly to prevent the virus from spreading rapidly, with particularly extensive measures in the spring 2020 and the fall and winter 2020/21. In most countries, including Germany, these lockdowns also included the closure of schools. For children and their families, these times were very challenging. Many parents experienced a triple burden of work, childcare, and homeschooling. Children had to stay at home and forgo contact with their peers. In addition, homeschooling imposed challenges by the requirements of higher self‐discipline and concentration abilities. It could also lead to an increase in conflicts between parents and children. Potential negative effects of school closures and social isolation on (mental) health, as well as on behavior and schooling, were discussed early on (Golberstein et al., 2020).

Regarding mental health, several longitudinal studies compared the wellbeing of children and adolescents before the pandemic and during the beginning of the pandemic/first lockdown in spring 2020. They showed an increase in depression (Bignardi et al., 2020; Zhang et al., 2020), psychological stress (Paschke et al., 2021), other mental health problems (Conti et al., 2020; Hussong et al., 2021; Ravens‐Sieberer et al., 2021), and behavioral difficulties (Achterberg et al., 2021; Conti et al., 2020; Hu & Qian, 2021), and a decreased quality of life (QoL; Ravens‐Sieberer et al., 2021). In our own preliminary work, we also showed a lower QoL during the first lockdown than before the pandemic (Vogel et al., 2021). Risk factors for reduced mental health are lower socio‐economic status (SES; Conti et al., 2020; Hu & Qian, 2021; Paschke et al., 2021; Ravens‐Sieberer et al., 2021; Vogel et al., 2021), family stress (Achterberg et al., 2021; Larsen et al., 2021), lack of peer support (Larsen et al., 2021; Mitra et al., 2021), and limited emotion regulation strategies (Paschke et al., 2021).

Concerning leisure behavior, previous longitudinal or retrospective studies, including our own preliminary work, showed longer screen times during the first lockdown than before the pandemic (Pietrobelli et al., 2020; Pombo et al., 2021; Vogel et al., 2021; Xiang et al., 2020). Regarding physical activity, several previous studies suggest a decrease during the lockdown (Eyler et al., 2021; Pietrobelli et al., 2020; Pombo et al., 2021; Xiang et al., 2020; Yomoda & Kurita, 2021). With respect to other leisure activities, own preliminary work showed that the frequency of interactive activities (handicrafts, board games) decreased from the beginning to the end of the first lockdown in spring 2020, suggesting that the duration of an exceptional situation may affect these activities negatively (Poulain et al., 2021).

Regarding possible effects of school closures or homeschooling on children's education, a large European study revealed that about 20% of parents were dissatisfied with the quality of homeschooling material and about 40% of them lacked support from schools or teachers during the first school closures in spring 2020, especially if their children had mental health problems (Thorell et al., 2021). Two longitudinal studies in elementary students, including own preliminary work, showed that children were enthusiastic about doing their schoolwork, but that motivation (Poulain et al., 2021) and completion of homework and online courses (Cui et al., 2021) declined from the beginning of the first lockdown to 1 month later.

All of the studies presented here were conducted in Europe, North America, or Asia; studies on wellbeing, health behavior, or homeschooling in lower‐middle income countries in Africa or Asia are sparse. However, recent reviews suggest that the challenges to mental health and education during the pandemic are particularly strong in these countries (Kar et al., 2020; Kola et al., 2021; Spaull & van der Berg, 2020; Yukich et al., 2021).

Although several longitudinal studies compared mental health before and during the pandemic, they mainly concentrated on the initial pandemic (spring 2020). Studies investigating children's wellbeing and behavior later in the pandemic are sparse. One longitudinal study conducted in the United States examined trends in adolescents' mental health from spring to summer 2020 (Hawes et al., 2021). It showed a peak in depression and anxiety symptoms in March 2020, followed by a decline through summer 2020. This result suggests that mental health might be particularly poor at pandemic peaks, but improve as the infectious situation improves. However, as this study looked at a short period including only one pandemic peak, it remains unclear how mental health would develop during further pandemic peaks.

The present study assessed and compared wellbeing, leisure behavior, and coping with homeschooling during two pandemic periods, both characterized by high infection rates and, subsequently, harsh anti‐pandemic measures, including school closures (lockdown in March 2020 and lockdown in January 2021). Regarding wellbeing, we compared the data with data collected in the year before the pandemic. Based on previous studies, we expected that children and adolescents would suffer more during renewed school closures (second lockdown) than during the initial school closure (first lockdown). Therefore, we hypothesized wellbeing, coping with homeschooling, and physical activity to be lower and media use to be higher during the second than during the first lockdown. Regarding SES effects, we expected wellbeing and coping with homeschooling to be lower in children from lower social strata. Furthermore, the decrease in wellbeing and coping with homeschooling was hypothesized to be stronger in these children.

## MATERIALS AND METHODS

### Participants

Data were collected within the LIFE Child study, a cohort study conducted in the city of Leipzig (Germany) investigating healthy development from prenatal stage to young adulthood (Poulain et al., 2017). Study participants are recruited through advertising in hospitals, public health facilities, and schools and come mainly from the city of Leipzig and surrounding areas. The LIFE Child study was designed in accordance with the ethical standards as laid down in the 1964 Declaration of Helsinki and its later amendments and approved by the Ethics Committee of the Medical Faculty of the Leipzig University (Reg. No. 264/10‐ek). Parents of all study participants provided informed written consent before the participation of their children.

Children who took part in the LIFE Child study before the pandemic were invited to complete online surveys on feelings and attitudes regarding the COVID‐19 pandemic at several time points in 2020 and 2021. For the present project, we analyzed survey data on wellbeing, homeschooling, and leisure behavior of 9‐ to 16‐year‐old children, assessed during the first German lockdown in March 2020 (t1) and during the second German lockdown in January 2021 (t2). Personalized links to the online surveys were sent by mail to 560 children at t1 and 755 at t2. Of those children, 276 (49%) and 285 (36%) completed the survey at t1 and t2, respectively. Only those children completing the survey at both t1 and t2 were eligible for the present analyses. This sample comprised 152 9‐ to 16‐year‐old children (55% of those who had completed the survey at t1). Due to missings in single survey items, the final analyses on wellbeing, coping with homeschooling, and leisure behavior were performed in subsamples of 117 (60 male, mean age = 12.6), 128 (65 male, mean age = 12.3), and 134 children (70 male, mean age = 12.5).

For the analyses of changes in wellbeing, data at t1 and t2 were completed by data collected before the pandemic, during the last regular visit in the LIFE Child study in 2019 (t0). Furthermore, we created a historical control group of LIFE Child participants who had provided information on wellbeing in the 2 years before the pandemic, that is, in 2018 and 2019. The controls were chosen matched (optimal matching algorithms) by age and sex with a case:control ratio of 1:3 (456 controls). The standardized mean age difference between the study sample and the control group was 0.0027 years. The variance ratio was 1.0021. Sex was matched exactly for all cases.

### Measures

Wellbeing was assessed using the physical wellbeing, psychological wellbeing, and peer and social support scales of the KIDSCREEN‐27 questionnaire on health‐related QoL (Ravens‐Sieberer et al., 2007). As personal contact was prohibited during the survey periods, we added the words “including online and via phone” to the question of whether the child spent time with friends (peer and social support scale). Sum scores of each scale were t‐transformed as described in the KIDSCREEN manual (Ravens‐Sieberer et al., 2007). Coping with homeschooling was assessed by five questions (motivation, concentration, fun, mastering, fear of bad marks) rated on a five‐point Likert scale (see Table 1). Leisure behavior was assessed by three questions (indoor physical activity, TV, computer games) rated on a five‐point Likert scale (see Table 1). SES was assessed using a composite score combining information on parental education, occupation, and income (adapted to Lampert et al. (2014)). The score ranges from 3 to 21, with higher scores indicating higher SES. For descriptive purposes, this score was categorized as low, middle, or high, based on cut‐off points deduced from a large representative German sample (Lampert et al., 2014).

**TABLE 1:** Survey questions on coping with homeschooling and leisure behavior

	German (original)	English (translation)
Coping with homeschooling		
Concentration	Konntest Du dich in der letzten Woche gut auf Deine Schulaufgaben konzentrieren?	In the last week, were you able to concentrate well on your schoolwork?
	***Antwortoptionen:*** nie, selten, manchmal, oft, immer	***Response options:*** never, seldom, quite often, very often, always
Motivation	Konntest Du dich in der letzten Woche gut selbst motivieren, die Schulaufgaben zu erledigen?	In the last week, were you able to motivate yourself well to complete schoolwork?
	***Antwortoptionen:*** nie, selten, manchmal, oft, immer	***Response options:*** never, seldom, quite often, very often, always
Fun	Hast Du die Schulaufgaben in der letzten Woche gern gemacht?	In the last week, did you enjoy doing schoolwork?
	***Antwortoptionen:*** nie, selten, manchmal, oft, immer	***Response options:*** never, seldom, quite often, very often, always
Mastering	Kamst Du in der letzten Woche gut mit den Schulaufgaben zurecht?	In the last week, have you mastered your schoolwork?
	***Antwortoptionen:*** überhaupt nicht, ein wenig, mittelmäßig, ziemlich, sehr	***Response options:*** not at all, slightly, moderately, very, extremely
Fear of bad marks	Befürchtest Du, dass Deine Noten wegen der Schulschließung schlechter werden?	Are you afraid that your grades will drop because of the school closure?
	***Antwortoptionen:*** überhaupt nicht, ein wenig, mittelmäßig, ziemlich, sehr	***Response options:*** not at all, slightly, moderately, very, extremely
Leisure behavior		
TV time	Wie lange hast Du dich in der letzten Woche pro Tag durchschnittlich mit folgenden Dingen beschäftigt? Filme, Serien	How long did you spend on average per day during the last week on the following things? Movies, series
	***Antwortoptionen:*** gar nicht, 30 Minuten, 1–2 Stunden, 3–4 Stunden, >4 Stunden	***Response options:*** not at all, 30 min, 1–2 h, 3–4 h, >4 h
Computer playing time	Wie lange hast Du dich in der letzten Woche pro Tag durchschnittlich mit folgenden Dingen beschäftigt? Computerspiele	How long did you spend on average per day during the last week on the following things? Video games
	***Antwortoptionen:*** gar nicht, 30 Minuten, 1–2 Stunden, 3–4 Stunden, >4 Stunden	***Response options:*** not at all, 30 min, 1–2 h, 3–4 h, >4 h
Indoor physical activity	Wie oft bist Du in der letzten Woche folgenden Tätigkeiten nachgegangen? Sport drinnen	How often did you perform the following activities in the last week? Indoor sports
	***Antwortoptionen:*** mindestens 1x/Tag, mindestens 3x/Woche, mindestens 1x/Woche, seltener als 1x/Woche, nie	***Response options:*** at least 1x per day, at least 3x per week, at least 1x per week, less than 1x per week, never

### Statistical analysis

Analyses were performed using R version 4.0 (R Core Team, 2017). Categorical and ordinal data were described as frequencies and percentages. Continuous data were described as means and ranges. Differences in wellbeing, coping with homeschooling, and leisure behavior (as dependent variables) depending on time, SES, or interactions between both (as independent variables) were investigated using multiple linear (Bates et al., 2014) or ordinal mixed‐effect models (Christensen, 2019), with the subject included as random effect. Effects were presented as non‐standardized regression coefficients (beta) or odds ratios (OR) and corresponding 95% confidence intervals (CIs). Interactions between time and SES were only included if the interaction term reached statistical significance (*p* < .05). Analyses were adjusted for the child's gender and age.

In the historical control group, differences in wellbeing between the two years before the pandemic (2019 and 2018) were assessed using paired *t*‐tests.

## RESULTS

The sample comprised 152 children aged 9–16 years who had completed the survey at both t1 (first lockdown) and t2 (second lockdown). Of these children, only 3% grew up in low SES families, while the majority grew up in families with either middle SES (52%) or high SES (45%).

**FIGURE 1:**
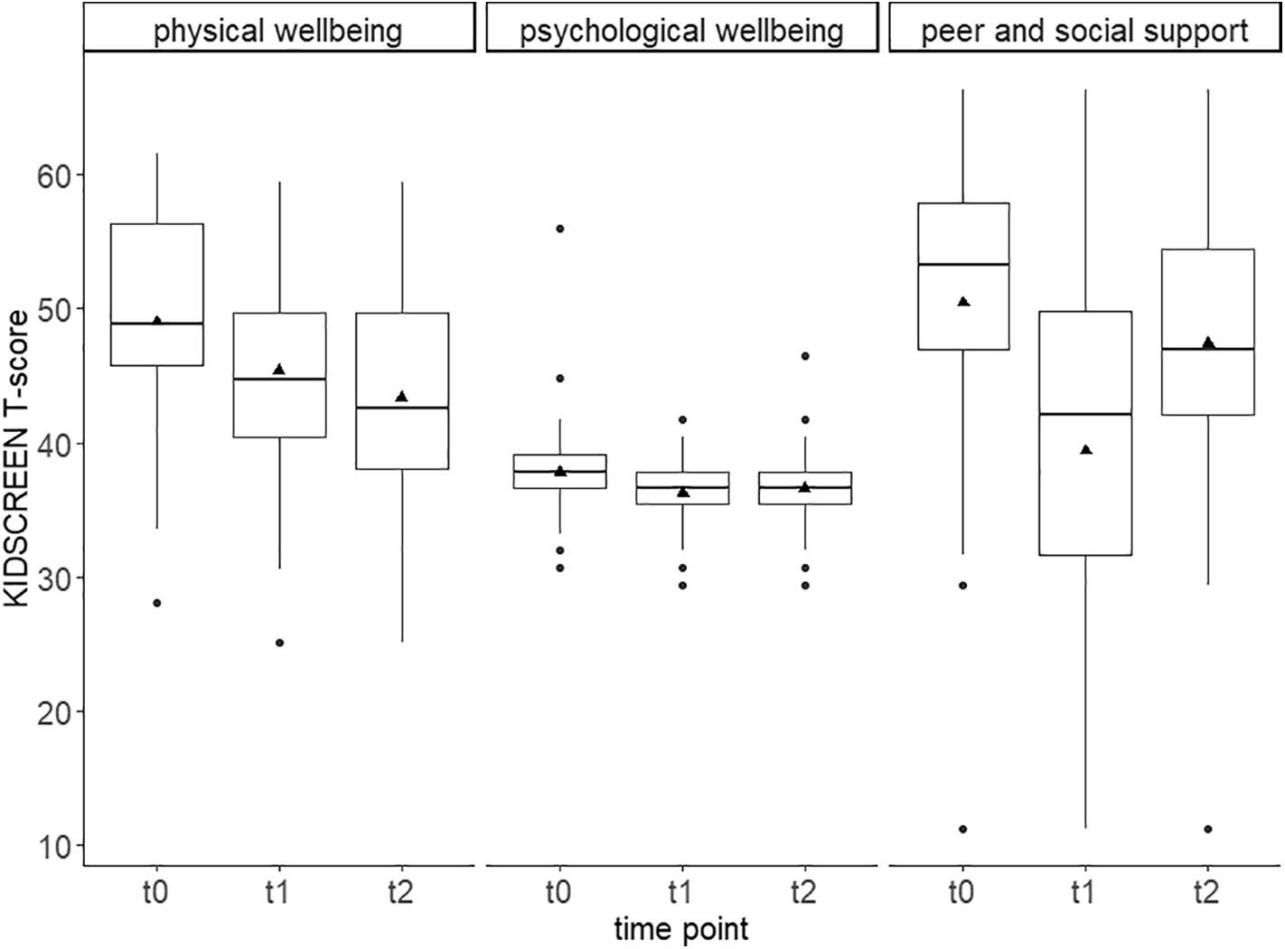
Physical wellbeing, psychological wellbeing, and peer and social support before the Covid‐19‐pandemic (t0), during the first lockdown in spring 2020 (t1), and during the second lockdown in winter 2021 (t2). The triangles indicate the mean t‐scores

Regarding wellbeing (*n* = 117), the mean KIDSCREEN t‐scores at t0 indicate average physical wellbeing (mean = 49.0, range 28–62) and average social support (mean = 50.4, range 11–66) before the pandemic, when compared to the reference population of the same age. Psychological wellbeing, in contrast, was rather low (mean = 37.8, range 31–56), even before the pandemic (t0). Our analyses revealed that physical wellbeing (beta = −3.6 [−4.9, −2.3], *p* < .001), psychological wellbeing (beta = −1.6 [−2.1, −1.0], *p* < .001) and social support (beta = −11.1 [−13.6, −8.5], *p* < .001) were significantly lower at t1 (first lockdown) than at t0 (before pandemic). Physical wellbeing further decreased between t1 and t2 (second lockdown, beta = −1.8 [−3.2, −0.5], *p* = .006), while we observed no significant change between t1 and t2 for psychological wellbeing (beta = 0.4 [−0.1, 1.0], *p* = .135). Social support, in contrast, increased significantly from t1 to t2 (beta = 7.7 [5.2, 10.3], *p* < .001), but was still significantly lower at t2 compared to t0 (beta = −3.3 [−6.0, −0.7], *p* = .015). These findings are illustrated in Figure 1. A higher SES was significantly associated with higher physical wellbeing (beta = 0.3 [.01, 0.6], *p* = .014), but not with psychological wellbeing (beta = 0.0 [−0.1, 0.1], *p* = .895) and social support (beta = 0.5 [−0.0, 1.0], *p* = .066). Also, the analyses revealed no significant interactions between SES and time (all *p* > .05).

In the historical control group, the mean KIDSCREEN scores were 48.9 (2018) and 48.7 (2019) for physical wellbeing, 37.8 (2018) and 38.2 (2019) for psychological wellbeing, and 52.9 (2018) and 53.6 (2019) for social support. None of the paired *t*‐tests revealed a statistically significant difference between 2018 and 2019 (*p* = 0.490, 0.095, and 0.210 for physical wellbeing, psychological wellbeing, and social support). In summary, the analyses showed that wellbeing did not change significantly in the years before the pandemic, while it decreased significantly during the pandemic.

**FIGURE 2:**
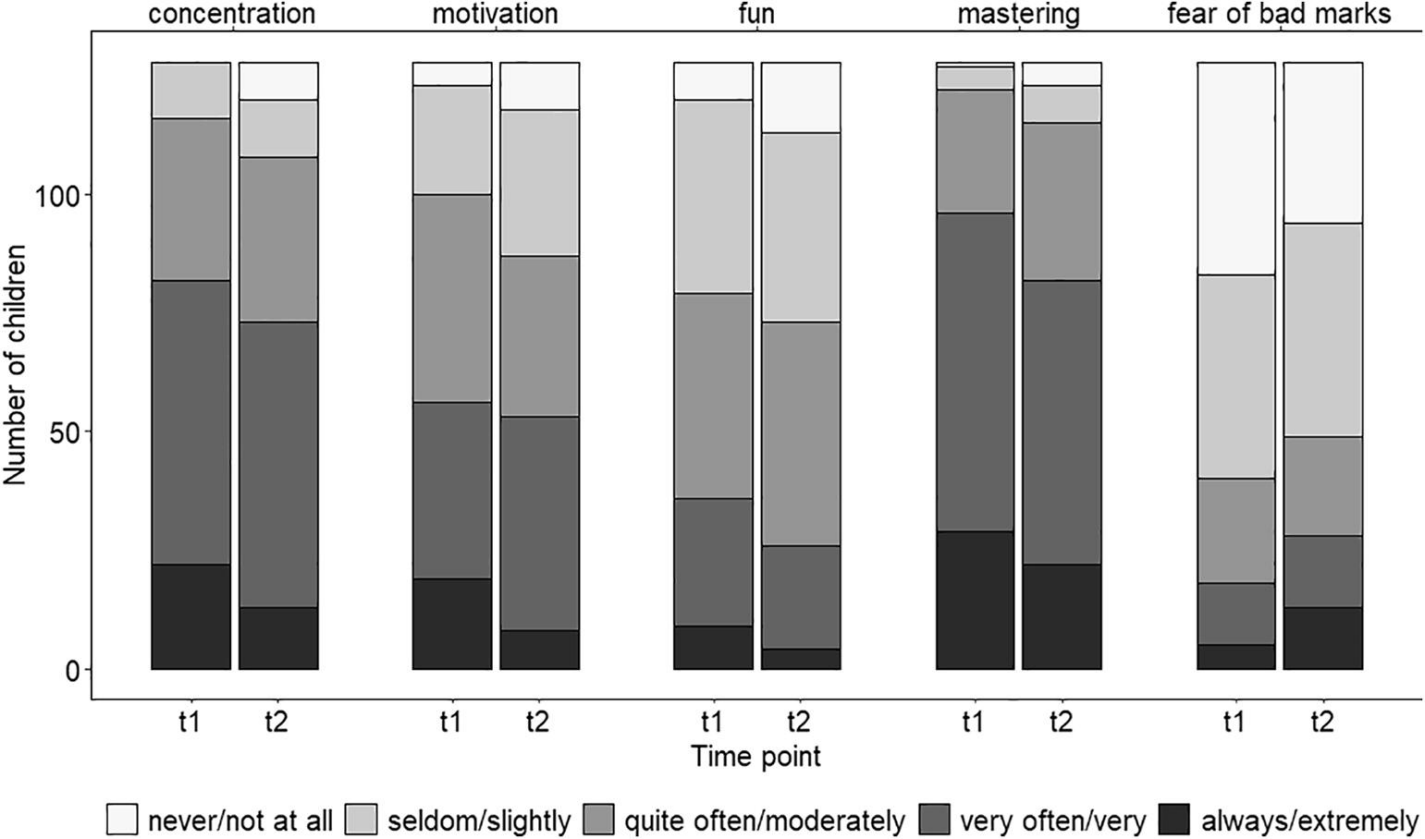
Coping with homeschooling during the first lockdown in spring 2020 (t1) and during the second lockdown in winter 2021 (t2)

With respect to coping with homeschooling (*n* = 128), most children reported being able to concentrate, being motivated, and having fun quite often or very often (see Figure 2). Also, the majority reported mastering schoolwork and having no fear of bad marks (see Figure 2). However, the analyses revealed significant changes in all items between t1 (first lockdown) and t2 (second lockdown). Concentration (OR = 0.5 [0.3, 0.8], *p* = .006), motivation (OR = 0.5 [0.3, 0.9], *p* = .012), fun (OR = 0.6 [0.4, 0.9], *p* = .024), and mastering (OR = 0.5 [0.3, 0.9], *p* = .011) were significantly lower at t2 than at t1, while fear of bad marks was higher at t2 than at t1 (OR = 1.9 [1.2, 3.0], *p* = .007). Figure 2 illustrated these differences. A higher SES was associated with higher concentration (OR = 1.2 [1.1, 1.4], *p* < .001), higher motivation (OR = 1.3 [1.1, 1.4], *p* < .001), more fun (OR = 1.2 [1.0, 1.5], *p* ≤ .001), better mastering of schoolwork (OR = 1.3 [1.1, 1.4], *p* < .001), and lower fear of bad marks (OR = 0.9 [0.8, 0.9], *p* = .003). Furthermore, there was a significant interaction between SES and time for the outcome concentration, indicating that the decline in concentration between t1 and t2 became significantly stronger as SES decreased (OR = 1.3 [1.1, 1.5], *p* = .004).

Regarding leisure behavior (*n* = 134), the analysis revealed no significant changes between t1 and t2 (all *p* > .05). Most participants reported watching films/series about 1–2 h per day (49% at t1, 47% at t2), playing computer games a maximum of 30 min per day (50% at t1, 48% at t2), and being physically active indoors at least three times per week (46% at t1, 39% at t2). Across all time points, a higher SES was significantly associated with less time spent playing computer games (OR = 0.9 [0.7, 0.98], *p* = .026). Associations between SES and TV time and physical activity were not significant (all *p* > .05). Also, no significant interaction between SES and time was observed for any outcome (all *p* > .05).

## DISCUSSION

This longitudinal study assessed wellbeing, coping with homeschooling, and leisure behavior of 9‐ to 16‐year‐old healthy children during the first and second COVID‐19‐related lockdowns in spring 2020 and in winter 2021. Schools were closed in both periods, so children had to do schoolwork at home. In addition, wellbeing was compared with wellbeing before the pandemic.

In line with previous findings (Achterberg et al., 2021; Bignardi et al., 2020; Conti et al., 2020; Hu & Qian, 2021; Hussong et al., 2021; Paschke et al., 2021; Ravens‐Sieberer et al., 2021) and our own preliminary work (Vogel et al., 2021), physical and psychological wellbeing as well as peer and social support were significantly lower during the first lockdown than before the pandemic. Given that wellbeing and social support had not changed significantly in the two years before the pandemic (2018–2019), these differences are likely to be explained by the pandemic or anti‐pandemic measures, for example, school closures, which might negatively affect the wellbeing of children and adolescents. Most interestingly, the analyses revealed a further significant decline in physical wellbeing from the first to the second lockdown, suggesting that children and adolescents do not adjust to the pandemic over time but that renewed school closures further weaken their already reduced physical wellbeing. Regarding psychological wellbeing, however, we did not find a significant change from the first to the second lockdown, and for peer and social support, we even observed an increase (without reaching the initial level). These findings might be explained by less rigid social restrictions during the second than during the first lockdown. During the first lockdown, one was not allowed to meet people from other households, whereas during the second lockdown, it was possible. Moreover, during the second lockdown, children up to the age of 12 were fully excluded from contact restrictions. With more personal contacts, peer and social support likely suffered less during the second than during the first lockdown. In addition, social support may have prevented further deterioration in psychological wellbeing.

Similar to (physical) wellbeing and in line with our expectations, coping with homeschooling decreased significantly from the first to the second lockdown. While concentration, motivation, fun, and mastering of schoolwork were lower during the second than the first lockdown, fear of bad marks showed a significant increase. In a previous study comparing concentration, motivation, and fun at two time points during the first lockdown (beginning and end, approximately 4 weeks apart), only motivation showed a significant decrease (Poulain et al., 2021). Taken together, the findings of both studies indicate that coping with homeschooling declined more from the first lockdown to the next than within the first lockdown. This suggests that children's frustration in returning to learning at home after temporary school openings is very high.

In contrast to wellbeing and coping with homeschooling, indoor physical activity, TV time, and time playing computer games did not change significantly between the first and the second lockdown. These leisure activities might represent robust behaviors exhibited with equal frequency in comparably challenging situations. In addition, these activities might be regulated by rules imposed by parents (e.g., regarding maximal screen times).

Regarding possible effects of familial SES on wellbeing, coping with homeschooling, and leisure activities, our hypotheses could largely be confirmed. As expected, children from socially disadvantaged families showed lower physical wellbeing, did less well with homeschooling, and played computer games more frequently than children from families with a higher SES. Furthermore, the decline in concentration from the first to the second lockdown was significantly stronger in children from families with lower SES. This finding strengthens the assumption that school closures have a particularly negative impact on this vulnerable group (Golberstein et al., 2020). However, contrary to our hypotheses and previous findings (Conti et al., 2020; Paschke et al., 2021; Ravens‐Sieberer et al., 2021), changes in wellbeing and other facets related to homeschooling did not differ significantly depending on SES. This may be explained by the fact that children from low SES families were underrepresented in this study (3%).

### Strengths and limitations

The present study investigated several aspects of children's health and behavior relevant in challenging periods like pandemic‐related school closures. Furthermore, we compared children's wellbeing at three time points before and during different periods of the pandemic and created a historical control group to show variations (or better said consistencies) in the years before the pandemic. Despite these clear strengths, some limitations have to be mentioned. First, the sample size was small and not representative in terms of SES (underrepresentation of low SES) and residency (underrepresentation of rural area). Therefore, generalizations to the entire population of children and adolescents are limited. Another limitation is that we did not assess information on wellbeing, coping with schoolwork, or leisure behavior between lockdowns, that is, when children went to school. Therefore, it cannot be excluded that the observed differences between first and second lockdown can be explained simply by the duration of the pandemic and the general restrictions (independent of school closures).

## CONCLUSION

The present findings suggest that renewed school closures after interim openings have a negative impact on physical wellbeing and coping with homeschooling. They, furthermore, suggest that less strict social restrictions might counteract a deterioration of psychological wellbeing and perceived social support. Therefore, anti‐pandemic measures such as school closures and contact prohibitions should be weighed against these negative effects and delayed as long as possible. If school closures are unavoidable in the future, tailored distance learning must be established, and children and their parents must be supported according to their individual needs. Complete restrictions on contact should be refrained from, as social contact with peers is particularly important in challenging times. Regardless of school closures, the range of support services for children and families in need must be expanded and simplified in order to prevent or counteract the development of serious mental disorders.

## CONFLICT OF INTEREST

The authors declare that they have no competing interests.

## ETHICAL CONSIDERATIONS

The LIFE Child study was approved by the Ethics Committee of the Medical Faculty of the Leipzig University (Reg. No. 264/10‐ek).

## AUTHOR CONTRIBUTION

**Tanja Poulain:** Conceptualization; Formal analysis; Methodology; Visualization; Writing – original draft; Writing – review & editing. **Christof Meigen:** Conceptualization; Data curation; Investigation; Software; Writing – review & editing. **Wieland Kiess:** Conceptualization; Funding acquisition; Project administration; Resources; Supervision; Writing – review & editing. **Mandy Vogel:** Conceptualization; Formal analysis; Methodology; Supervision; Visualization; Writing – review & editing.

## PATIENT CONSENT STATEMENT

All participants were informed on the study content and provided informed written consent before participation in the LIFE Child study.

## PERMISSION TO REPRODUCE MATERIAL FROM OTHER SOURCES

Not applicable.

## CLINICAL TRIAL REGISTRATION

Not applicable.

## Data Availability

Data of the LIFE Child study cannot be shared publicly because the data contains potentially sensitive information and publishing data sets is not covered by the informed consent provided by the study participants. Researchers interested in accessing and analyzing data collected in the LIFE Child study may contact the data use and access committee (forschungsdaten@medizin.uni-leipzig.de).
